# Adaptations to HIV services delivery amidst the COVID-19 pandemic restrictions in Kampala, Uganda: A qualitative study

**DOI:** 10.1371/journal.pgph.0000908

**Published:** 2022-08-23

**Authors:** Jonathan Izudi, Agnes N. Kiragga, Stephen Okoboi, Francis Bajunirwe, Barbara Castelnuovo

**Affiliations:** 1 Infectious Diseases Institute (IDI), Makerere University College of Health Sciences, Kampala, Uganda; 2 African Population and Health Research Center, Nairobi, Kenya; 3 Department of Community Health, Faculty of Medicine, Mbarara University of Science and Technology, Mbarara, Uganda; The University of Texas Health Science Center at Houston School of Public Health - San Antonio Campus, UNITED STATES

## Abstract

The enforcement of the coronavirus disease (COVID-19) pandemic restrictions disrupted health services delivery and currently, there is a limited understanding regarding measures employed by health facilities to ensure delivery of human immunodeficiency virus (HIV) services amidst the interruptions. We, therefore, designed a qualitative study to explore the measures for continuity of HIV services during the COVID-19 pandemic restrictions in Kampala, Uganda. This study was conducted at six large primary health care facilities in the Kampala Metropolitan area. Qualitative data were collected from anti-retroviral therapy (ART) focal persons and lay health workers namely linkage facilitators and peer mothers through key informant interviews (KIIs). Overall, 14 KIIs were performed, 10 with lay health workers and 4 with ART focal persons. Data were audio-recorded, transcribed verbatim, and analyzed using the content approach, and the results were presented as themes along with participant quotations. Five themes emerged to describe measures for continuity of HIV services. The measures included: 1) leveraging the use of mobile phone technology to support ART adherence counseling, psychosocial care, and reminders concerning clinic appointments and referrals; 2) adoption of novel differentiated service delivery models for ART like the use of motorcycle taxis and introduction of an individualized ART delivery model for patients with non-disclosed HIV status; 3) scale-up of existing differentiated service delivery models for ART, namely multi-month dispensing of antiretroviral drugs (ARVs), fast-track ARV refill, home-based ARV refill, peer ART delivery, use of community pharmacy model, and community client-led ART delivery model; and, 4) reorientation of health facility functioning to the COVID-19 pandemic restrictions characterized by the use of nearby health facilities for ARV refill and viral load monitoring, transportation of healthcare providers and flexible work schedules and reliance on shift work. We found several measures were adopted to deliver HIV care, treatment, and support services during the COVID-19 pandemic restrictions in Kampala, Uganda. We recommend the scale-up of the new measures for service continuity in the post-COVID-19 period.

## Introduction

Between March 18 and June 14, 2021, Uganda enforced several restrictions, namely night-time curfews, travel bans, school closure, and physical and social distancing requirements that constituted a national lockdown to prevent the spread of the coronavirus disease (COVID-19) pandemic [1]. These restrictions disrupted the delivery of maternal, neonatal, child, sexual and reproductive health services [2], human immunodeficiency virus (HIV) [3], tuberculosis [4], and mental health [5], including disruptions in the country’s socio-economic development [6–8]. The disruptions in the health sector included the re-deployment of the existing health workforce to respond to the pandemic, difficult access to health facilities by patients, and a shift in healthcare priority to pandemic control and prevention [9].

To mitigate the negative impact of such disruptions on the delivery of essential health services such as HIV, the World Health Organization (WHO) recommended that countries enforce measures to ensure continuity of health services delivery amidst the disruptions [9]. Subsequently, the Uganda Ministry of Health developed and disseminated guidelines for the continuity of essential health services to direct the delivery of HIV services at both national and sub-national levels, with a focus on reducing the vulnerability of people living with HIV (PLHIV) to COVID-19, ensuring continuity of HIV treatment, prevention, screening, and diagnostic services, and minimizing the transmission of COVID-19 [10]. However, these guidelines did not describe specific measures that health facilities could deploy for the continued provision of HIV services. There was also no guidance on how existing health service delivery models should be modified to ensure continuity of HIV service delivery. However, anecdotal observations indicate that several measures to HIV services delivery were adopted by health facilities including modifications in existing HIV service delivery models, which have largely not been rigorously documented and reported.

Existing evidence regarding the effect of the COVID-19 pandemic restrictions on the delivery of HIV services in Uganda is mixed. Data from the Presidential Emergency Plan for AIDS Relief (PEPFAR) show no changes had occurred in viral load testing and viral load suppression rates during the restrictions [11], whereas another study reported an improvement in viral load testing and suppression [12]. Among PLHIV/hypertension, one study reported nearly universal access to HIV medicines during the restrictions [13] and another study reported a decline in retention [14].

The lack of effect of the restrictions on HIV service delivery or improvements in others could be explained by either novel measures to HIV service delivery or modifications in existing service delivery models. However, there is limited documentation of the strategies adopted by health facilities to ensure uninterrupted delivery of HIV services during the COVID-19 pandemic restrictions, including modifications in previous HIV service delivery measures. Therefore, we explored the measures for continuity of HIV services delivery during the COVID-19 pandemic restrictions in Kampala, Uganda from the perspectives of health workers who provide HIV care, treatment, support, and prevention services at the health facility and community levels. This information has the potential to inform the national scale-up of measures that have proved effective in ensuring continuity in HIV service delivery and achieving better patient outcomes amidst the COVID-19 pandemic restrictions in Uganda.

## Methods and materials

### Study design and setting

We conducted a qualitative study to enable a description and an in-depth understanding of measures used to ensure continuity of HIV services. The study was conducted at the six Kampala Capital City Authority (KCCA) health facilities, namely Kisenyi Health Center IV (HC IV), a county-level health facility, and the other health facilities, namely Kawaala, Kisugu, Kitebi, Komamboga, and Kiswa, all sub-county level health facilities. According to Uganda’s healthcare system, a county-level health facility provides basic preventive, curative, and rehabilitative care as well as referral services for life-saving medical, surgical, and obstetrical emergency care such as blood transfusion, caesarean section, and other medical and surgical emergency interventions to a catchment population of 100,000 people [15]. Conversely, a sub-county level health facility provides basic preventive, promotive, and curative care to 20,000 people as well as technical support to lower-level facilities. All sub-county level health facilities also provide some basic laboratory/diagnostic services, maternity care, and first-level referral care [15]. All the selected KCCA health facilities have an HIV clinic which is managed by an ART focal person who is either a medical doctor, clinical officer, or nursing officer. Each study site has a linkage facilitator and a peer mother(s). The linkage facilitator provides community and between versus within health facility linkages for patients after a positive HIV test. Notable roles include documenting patient contacts and referrals, acquisition of consent for home visits, and tracking patient referrals to other health facilities.

Peer mothers strengthen patient counseling and education services at the health facilities by providing standardized patient counseling services, including adherence and psychosocial support. Overall, HIV care is provided per the 2020 Uganda National HIV Treatment guideline [16].

### Study participants

The participants included ART focal persons, linkage facilitators, and peer mothers sampled purposively because they were deemed knowledgeable about the delivery of HIV care. ART focal persons provide HIV clinical care, support, and prevention services to PLHIV. Mostly, they are the heads of the HIV clinic and are responsible for the management functions of the clinic. Linkage facilitators are lay healthcare workers who connect individuals who have tested positive for HIV from one service point to another. Linkage is deemed successful when the person is enrolled in HIV care and treatment. The timeline for linkage is seven days if within the same health facility, otherwise 30 days for inter-facility or community-facility linkage. Linkage facilitators also connect those with a negative HIV test result to appropriate HIV prevention service packages. Peer mothers are lay healthcare providers trained to provide standardized patient counseling services including adherence and psychosocial support. We included participants who had worked at the HIV clinic during the enforcement of the COVID-19 pandemic restrictions. These criteria ensured that we interviewed participants who were familiar with measures employed to ensure continuity of HIV care services at the respective health facilities. We excluded participants who were transferred in at the health facility after the enforcement of the restrictions. We deemed such participants do not have a clear understanding of measures employed to deliver HIV services during the restrictions.

### Variables and measurements

The interviews focused on four major topics: 1) *HIV services orientation*: we explored measures taken by health facilities to ensure access to HIV care. We probed the measures for HIV testing, linkage into care for those newly diagnosed with HIV, retention, access to HIV viral load testing, and specific measures for children, adolescents, and adults; 2) *Community and patient-centered HIV services delivery models*: we explored measures for ART delivery, new measures for ongoing psychosocial and ART adherence support, and measures for sick patients who need medical care or referral for admission; 3) *Staff alignment or re-deployment to ensure service continuity*: we probed the changes that were implemented concerning the organizing of staffing at the health facility and community levels, changes in staff deployment and how it affected the delivery of HIV services, and changes in staff roster/schedules and how it affected service delivery; 4) *Modifications in the organization and delivery of HIV supplies and commodities*: we probed the changes that were implemented to ensure a consistent supply of ARVs and HIV test kits among others, the monitoring of stock levels, changes in the ordering and distribution/re-distribution of HIV commodities or supplies and new measures to dispensing of HIV medications.

### Data collection

Qualitative data were collected through key informant interviews (KIIs) with the selected individuals at the respective health facilities (S1 File). All the interviews were conducted in the English language within the health facility premise between February and March 2022, Monday to Friday, 10.00 am to 5.00 pm. Two interviewers (NN and JI) conducted the interviews. NN conducted and moderated the interviews while JI took field notes. NN is a female social scientist, with a BA in Social Sciences and 7 years of experience in qualitative research. JI is a male public health specialist, with a doctoral degree and 5 years of experience in mixed methods research, largely in infectious diseases. Each interview lasted 20 minutes on average.

### Data analysis

We depended on the saturation principle, a point when further interviewing of participants provided no new information, to reach the required sample size. We stopped the interviews after attaining the saturation point.

Regarding data processing, the audio-recorded responses were transcribed verbatim within 24–48 hours, verified by re-playing and reading through the transcripts and any discrepancies were identified and corrected. The transcripts were imported into R version 4.0.2 for content analysis using an inductive approach. We used the *R Qualitative Data Analysis (RQDA)* package for the analysis, which was independently performed by two Data Analysts (JI and NN) to prevent subjective bias. Each data analyst coded the transcripts and developed a codebook, which was later harmonized to generate the final codebook (S2 File).

We used an inductive thematic content analysis approach that followed three steps, namely data immersion, coding, and coding sort. In data immersion, two analysts (NN, a female qualitative researcher with 7 years of research experience, and JI, a male public health specialist with 5 years of experience in mixed-methods research) familiarized themselves with the transcripts by reading and re-reading them several times to identify common and important texts. The analysts (NN and JI) allowed impressions to shape interpretations in different and unpredicted directions [17]. During the coding stage, NN and JI flagged relevant parts of the transcripts with suitable codes and in the third stage (coding sort), NN and JI sorted the codes and transformed them into themes/sub-themes. Two other reviewers (BC and AK) verified all the codes and the themes/sub-themes. BC and AK are female research scientists with immense experience in quantitative and qualitative research. Each theme was presented with quotation(s) from the participants to contextualize the findings along with unique identifiers.

### Quality control measures

The codes and themes were verified by two other analysts (BC and AK). Research Assistants received a 2-day training on the study protocol and responsible conduct of research, including participant sampling and questioning techniques. All audio-recorded responses were transcribed within 24–48 hours and field notes were summarized within 24 hours to ensure data accuracy. The data collection process was supervised by a Field Supervisor (SO).

### Ethical considerations

Our study received ethical approval from the Infectious Diseases Institute Research Ethics Committee (reference number 013/2021) and the Uganda National Council for Science and Technology (reference number HS709ES). We received administrative approval from the Directorate of Public and Environmental Health of Kampala Capital City Authority (reference number DPHE/KCCA/1301). All the participants provided written or thumbprint informed consent after receiving information about the study. The specific information included the study rationale, objectives, potential benefits and risks, why a participant was deemed appropriate, and withdrawal if any. We ensured voluntary participation in the study and withdrawal at any time if one wished to do so.

### Reporting of findings

We adhered to the consolidated criteria for reporting qualitative studies (COREQ) guideline, a 32-item checklist, in reporting our findings [18].

## Results

### Participant characteristics

Table 1 summarises the characteristics of the participants in the study: ART focal persons versus lay healthcare providers (linkage facilitators and peer mothers). We held interviews with 14 participants. The participants were largely from Kitebi and Komaboga HC IIIs and an equal number of linkage facilitators and peer mothers were interviewed. The majority of the participants were aged 30–34 years, were female, had attained university-level education, and were married. On average, the interviews lasted for 24 minutes.

**Table 1:** Participant characteristics.

Variable	Level	Total (n = 14), frequency	ART focal person (n = 4), frequency	Lay health workers (n = 10), frequency
Health facility	Kawaala HC III	2	1	1
	Kisenyi HC IV	2	1	1
	Kisugu HC III	2	1	1
	Kiswa HC III	2	1	1
	Kitebi HC III	3	1	2
	Komamboga HC III	3	1	2
Age group (years)	30–34	9	3	6
	35–45	5	1	4
	Mean (SD)	34.4	33.3 (3.4)	34.9 (6.5)
Sex	Female	11	3	8
	Male	3	1	2
Level of education	Secondary	5	0	5
	Tertiary	3	0	3
	University	6	4	2
Marital status	Married	8	3	5
	Separated	1	0	1
	Single	5	1	4
Duration of interviews (minutes)	Mean (SD)	24.1	24.8 (5.3)	23.9 (11.1)

### Themes and sub-themes

Table 2 summarises the emergent themes and sub-themes. Five themes emerged from the study, namely leveraging the use of mobile phone technology for HIV care, adoption of novel differentiated service delivery models for ART delivery, scale-up of existing differentiated service delivery models for ART delivery, and reorientation of health facility functioning to the COVID-19 pandemic restrictions.

**Table 2:** Summary of emergent themes/sub-themes.

Themes	Sub-themes
Leveraging the use of mobile phone technology for HIV care	•Mobile-phone-based ART adherence support. •Mobile-phone-based psychosocial counseling. •Mobile-phone-based reminders about clinic appointments and referrals.
Adoption of novel differentiated service delivery models for ART delivery	•Use of motorcyclists to deliver ARVs. •Individualized ART delivery for patients with non-disclosed HIV status.
Scale-up of existing differentiated service delivery models for ART delivery	•Multi-month dispensing of ARVs. •Fast-track ARV refills. •Home-based ARV refill. •Peer ART delivery approach. •Use of community pharmacy •Use of Community Client-led ART delivery model.
Reorientation of health facility functioning to the COVID-19 pandemic restrictions.	•Use of nearby health facilities for ARV refill and viral load monitoring. •Transportation of health workers to health facilities and flexible work schedules. •Introduction of shift work.

### Theme 1: Leveraging the use of mobile phone technology for HIV care

This theme comprised of three sub-themes, namely the use of mobile phone technology for HIV care, treatment, and support functions during the COVID-19 pandemic restrictions.

#### Mobile-phone-based ART adherence support

ART adherence is the cornerstone of a successful HIV Control Program. Participants mentioned that PLHIV continued to receive ART adherence counseling during the COVID-19 pandemic restrictions through mobile phones and the priority was accorded to those with detectable viral load. New patients received face-to-face counseling, whenever they could reach the health facility.

*“We used to counsel them*. *It never stopped*. *The ones with high viral load we were counselling them on phone but for new patients who just tested*, *it was face to face counselling*. *We would counsel them immediately then we would start them on ART” (LF*, HF06).

*“For sure when it came to counselling*, *we used phone counselling*. *Yes*, *we used phone counselling because some people had viral load issues but couldn’t come at the facility in person*. *So we used phone counselling by then IDI (meaning the Infectious Diseases Institute) used to provide enough airtime*, *we could counsel them on phone” (LF*, *HF05*).

#### Mobile-phone-based psychosocial counselling

Mobile phones were used to provide general psychosocial counseling during the COVID-19 pandemic restrictions. Participants mentioned that patients who could not reach the health facility were contacted over the phone and then counseled.

*“We would make phone calls and we conduct an online counselling session*. *So we would get on phone and tell them the importance of adhering well*. *And the proper way of taking the medication through phone*. *We had them counselled through phone” (LF*, HF04).

*“We still gave them psychosocial support*, *especially to those ones who can come to the facility*. *Those ones who fail to come*, *we get their contacts and give the file to the counsellors then the counsellors speaks to them via phone wherever they are*. *The counsellor then indicates that in the file” (PM*, HF02).

#### Mobile-phone-based reminders about clinic appointments and referrals

To support adherence to clinic appointments, especially for PLHIV who had access to the health facilities during the COVID-19 pandemic restrictions, it emerged that healthcare providers used mobile phones. The clients were called and reminded of their exact next clinic appointment dates. The calls were made 2–3 days before the scheduled appointment date.

*“Another thing we used to do was to pre-call and remind them (PLHIV) about their appointments*. *So those who could afford used to come to the facility” (LF*, HF06).

*"Then*, *we the peer mothers had the responsibility of doing the pre-calling using phones*. *Some of the people can even forget to come to the clinic so we will call like three to four days before to tell them they are needed in the clinic*. *This way*, *they always came because they felt we care about their health” (PM*, HF03)

### Theme 2: Adoption of novel differentiated service delivery models for ART delivery

This theme focused on ensuring an uninterrupted supply of ARVs and other medications for PLHIV. Broadly, two new models emerged to support ART delivery and they included the use of motorcycle cyclists and an individualized ART delivery model for patients with non-disclosed HIV status.

#### Use of motorcyclists to deliver ARVs

The Government of Uganda banned the use of public and private transport means but allowed the use of motorcycles for the delivery of food items. The motorcycle taxis commonly referred to as "Boda boda" were contacted by healthcare providers and used to deliver ARVs and other medications to PLHIV who had difficult or no access to the health facilities.

*“During COVID time (meaning the COVID-19 pandemic restrictions)*, *we had the boda boda delivery model*, *which was not there before*. *It was introduced during that period*. *Boda boda model worked in a way that when a patient’s appointment date reached and they could not reach the facility because of the lockdown and the transport issues*, *IDI (Infectious Diseases Institute) came in with the model of boda boda*. *So we could just send the drugs to our patients to their homes*. *Because they were trying to limit the numbers so that we have few patients at a time” (LF*, HF02).

*“Then other thing that came on board was because the Boda Boda…*.. *Were still allowed to operate*. *We had the Boda Boda model*. *It was innovated by our supervisors*, *whereby we would generate a list after calling our patients who are within Kampala and Wakiso but had failed to make it to the health facility because of transportation means*. *Or maybe because transport was still expensive or whatever reason was associated to inaccessibility or difficulty accessing the facility*, *we would put them on a boda* boda list.

*So we would find out from their files about the drugs they are taking*, *their location or residence*, *confirm the location and then contact them during the pre-calling and tell them*, *“That your appointment is next week*. *Will you be able to make it*? *If not*, *are you okay with us delivering you the drugs by a boda boda*?*" (FP*, HF04).

#### Individualized ART delivery for patients with non-disclosed HIV status

The use of motorcyclists for home delivery of ARVs enables the continued supply of ARVs and other medications but was not universally welcomed by PLHIV especially those with non-disclosed HIV status. These patients would not allow healthcare providers to use motorcyclists to deliver ARVs to their homes or even conduct a home visit. Instead, the patients preferred to have their motorcyclist deliver ARVs and other medications, and this individualized ART delivery was accepted to meet their needs and convenience.

*“Some patients*, *you just have to understand them*. *They have too much stigma*. *They say*, *"You don’t send us your boda boda (meaning motorcylists to deliver ARVs)*. *I will get another boda boda and pay for it myself"*, *because he will not know what he is carrying” (LF*, HF05).

*“Some of the mothers*, *their husbands are not aware of their status*. *They have never disclosed to them*. *So when you talk to her she can tell you not to reach the home*. *Even most of them we know them*. *That this one doesn’t want us to reach home*. *Others used to tell us that*, *“clinician*, *when you reach this junction*, *you call me*. *This guy is around”*. *So you had to abide by that so that you save her marriage then you meet her where she tells you as long as it is a boda boad place” (PM*, HF03).

### Theme 3: Scale-up of existing differentiated service delivery models for ART delivery

Several known ART delivery models were scaled-up to ensure continued delivery of ARVs and they included: multi-month dispensing of ARVs, fast-track ARV refill, home-based ARV refill, peer ART delivery, and use of community pharmacy and community client-led ART delivery models. We describe these measures below.

#### Multi-month dispensing of ARVs

Healthcare providers maximized every opportunity they had with patients to sufficiently provide ARVs. It emerged that rather than the usual care of dispensing ARVs for 1–2 months, ARVs were dispensed for at least 3 months.

*“The quantity (meaning ARVs) increased because of course being a hard time for each one of us*, *for the patients and for the service providers*. *We tried as much as possible to fit in the shoes of the patient because they always came complaining that*, *"I may not be able to come back for medication next time"*. *So we tried to increase the quantity that we were giving out to the patients*. *We stopped giving out for one month*, *we now give for three months and above*. *The maximum number of months was five” (LF*,*HF*04*)*.

*“…*..*And for those ones who made it to the facility*, *we made sure we gave them a long refill because we didn’t know how long the pandemic would take*. *So we would give them at least three months*, *and that would take them for some time to avoid the inconvenience of coming back every month or every after two months unnecessarily*. *So*, *all patients who came*, *whether with a high viral load and were supposed to come back every after one month*, *we gave them as many pills as possible*. *We were not giving one month*, *we were giving three months because we didn’t know how long lockdown would take (FP*, HF04).

*“During the lockdown*, *the months for refiling ARVs [meaning anti-retroviral drugs] had to increase at least from 2–3 months or even more*. *Those not due for viral load testing were getting more than four months refill” [LF*, HFO2].

*“Now*, *because of the pandemic*, *when somebody [meaning person living with HIV] is suppressed*, *we put them on the 6 months ARV supply then those who are not suppressed*, *remain on one month*. *But most of them were suppressed so they were put on six months’ supply*. *If somebody is not suppressed*, *that means they need attention so those ones remained on one month until when they are suppressed*. *When they get suppressed*, *they get switched to six months” [LF*, HF01].

#### Fast-track ARV refills

The participants indicated that ARV refills were not based on a differentiated care delivery model. A fast-track ARV refill was employed to reduce the contact time between healthcare providers and patients and to serve patients as fast as possible. In this approach, the patients received ARV refills without any attention to the ART differentiated service delivery models because differentiating care would mean a longer waiting time at the health facility.

*“So all we were doing was refill and go*, *refill and go*, *refill and go*. *So at that time we were not differentiating care*. *So once you come in*, *we give you three months*, *you walk away*, *come in*, *three months*, *and walk away (*FP, HF04).

*"But then with time*, *when the expert clients [meaning experienced PLHIV] came back to work*, *we tried to make sure that we retrieved the patient files but we did it very fast*. *Sometimes we would do it [meaning file retrieval] even the day before*, *so that we reduce on delays among the patients during ARV refill" (*FP, HF02).

#### Home-based ARV refill

The majority of patients could not access the health facilities for medication refills and a few who could had fears due to stigma and discrimination. The patients feared that their HIV status would get known by the general public if they continued to visit the health facilities amidst the restrictions. For such patients, healthcare workers conducted home visits to deliver ARVs and other medications.

*“However*, *there are those ones that were like for me*, *I stay nearby but I cannot come to the health facility*. *So*, *what we (HIV Care Team) used to do was to get a locator form from the file and then try our level best to pick the drugs (meaning ARVs)*, *inform the clinician*, *then sign in the client’s file and then carry them (meaning the ARVs) to the client’s home*. *That’s how we used to do it*.*” (PM*, HF04).

*"We (peer mothers) were following them (PLHIV)*. *We were calling basing on their appointment dates then asking where they stay*. *We deliver to them drugs at home*. *For those ones who are nearby*, *we would also deliver the drugs to them*.*”* (PM, HF02).

#### Peer ART delivery approach

With difficult access to health facilities for the majority of the patients, a peer ART delivery model was introduced to improve access to ARVs. Here, expert clients (PLHIV dedicated to helping their peers) took ARVs and other medications to their peers who could not reach the health facilities.

*“So the other thing that we did here*, *we have what we call the volunteers or the peers in care*. *These are people who are also receiving treatment from our health facility*. *They are also in care and are from different parts of the town*. *We allowed them to take drugs to patients who came from within that area where these peers come from (stay) so long as they had the patient network*. *We would allow them to carry drugs for them*. *We would update the charts for the patients that they are taking drugs* for.

*“The expert clients would call the patients and if they were unable to come for the drugs*, *they would reach there and take for them the drugs” (FP*, HF04).

#### Use of community pharmacy

ART delivery in Uganda has been scaled to the community level under a differentiated service delivery model known as community drug distribution points, sites within the community where ARVs are distributed at patient convenience. During the pandemic restrictions, community ART delivery models were extensively used to reach where patients lived and worked.

*“So these patients when their return dates reaches*, *they go to the community pharmacy; those pharmacies that are in the community next to their homes*. *So those ones never had any problems getting their drugs and those who had problems*, *equally the boda boda program had to come in” (LF*, HF04).

*“We have clients here who get drugs from the community pharmacy*. *So those ones used to get drugs from the community pharmacy*. *Apart from the community pharmacy and boda boda*, *there was nothing else” (LF*, HF02).

#### Continued use of Community Client-led ART delivery (CCLAD) model

This was another approach to ART delivery within the differentiated service delivery model at the community level. Under this model, PLHIV within a certain residence formed a group that consisted of 6–10 people, with one person as a Team Leader. Each member was assigned to collect ARVs for the entire group on a rotational basis. It emerged that this model was used a lot during the COVID-19 pandemic restrictions.

*“We have the community Client-Led ART Delivery (CCLAD) model where the patients make a group by themselves and then they alternate in picking the drugs for the rest of the team*. *So*, *they make a group of six people who reside in the same community*. *But before they start their group*, *they first come here then you orient them in doing their work*, *on assessing each other for TB (meaning screening for tuberculosis)*. *Then in that group*, *we decide who will come first based on how their viral loads are*. *So we make sure that the person who comes to pick drugs for the whole group if they are due for bleeding*, *you bleed them*. *Now you give them appointments every two months*.*” (FP*, HF04).

*“So they make a group of six people who reside from the same community*. *But before they start their group*, *they first come here then you orient them in doing their work*. *Then in that group*, *we decide who will come first*. *Now you give them appointments every two months*. *You give them appointment every two months then they alternate*, *today it is patient A after two months it is patient B after two months it is patient C like that*. *So when they pick the drugs*, *they go back to the community and give to the other group members*. *But when patient A comes you retrieve files for all the group members*.*”* (HF01, LF).

### Theme 4: Reorientation of health facility functioning to the COVID-19 pandemic restrictions

Within the health facilities, several adjustments were implemented to enable the delivery of HIV care, treatment, and support amidst the COVID-19 pandemic restrictions. The reorientation of health facilities functioning to the COVID-19 pandemic restrictions included the following:

#### Use of nearby health facilities for ARV refill and viral load monitoring

During the restrictions, access to health facilities was difficult and when it became clear that a national lockdown would be enforced, most of the patients traveled to their villages, largely upcountry. Such patients had challenges in accessing ARVs including routine viral load monitoring. Participants reported that such patients were referred to access HIV services at the nearest health facility.

*“We had another system*. *Someone says*, *"Am very far*, *I went to the village"*. *We would advise such patients to go to the nearest hospital or health facility*. *Even us here*, *we were refilling patients whom lockdown found in Kampala and could not go back to their original health facilities where they had been getting treatment from*. *We would counsel them that*, *"you go to the nearest facility and they refill for you until they open up their transport or the lockdown” (LF*, HF05)

*“……We were calling some of them (meaning patients) and advising them to go to the health facilities near their residences*. *They would get a refill from there*, *and as soon as they get a refill*, *they communicate” (FP*, HF04).

*“Those ones who were not within our proximity*, *we would tell them to go to health facilities near them and we would call the other facility to ensure their retention” (LF*, HF04).

#### Revised workflow

Health facilities introduced new systems of work to ensure continuity of HIV services. For example, the use of appointment registers/systems in real-time was abandoned while new workspaces were created.

*“The client used to remain this side*, *not to go the other side*. *That is how we used to do*. *But it was not easy because me a linkage facilitator*, *that is where I sit*, *on that table so I used to put my chair on that side and for the client the other side (LF*, HF06).

*“So what we did is that we had a book in which we were writing patients who came in*. *We put aside issues to do with appointment register*. *In that book*, *we would write your file number*, *name*, *date you came and how much drugs you have been given and the regimen then we would use that book to update the patient’s* register later on.

*So we were not withdrawing files during that time*. *By doing that*, *we would spend very little time with the patient and would not have congestion*. *We would clear the patients very fast and they would leave” (FP*, HF04).

#### Transportation of health workers to health facilities and flexible work schedules

Healthcare workers were transported from their homes to health facilities to enable continuity of HIV care and then back. In instances where it was difficult to transport a healthcare provider to his/her health facility, they were advised to report and work at the nearest health facility.

*“We were coming everyday although the staff was reduced*. *Those ones coming from far could not come because of transport*. *The few who were supposed to come*, *there were those who would be picked and dropped here by car and then there were those who could not*.*”* (PM, HF02*)*.

*“We had a bit of flexibility*. *For example*, *during lockdown*, *because public transportation was not available*, *so some of the clinicians could not come to their places of work*. *IDI (meaning Infectious Diseases Institute) provided a van to take clinicians to their places of work but even then clinicians were told to go and work in health facilities near where they stay*. *If you are staying near Kisenyi*, *you would be told to go and work in Kisenyi*. *So we had a bit of changes in the roster” (*FP, HF02).

#### Introduction of shift work

Considering that the majority of healthcare providers use public transport means to reach workplaces, the number of healthcare providers who could reach the health facility significantly reduced during the restrictions due to transport challenges. To ensure continuity in the delivery of HIV services, shift work was introduced.

*“Yes*, *there were changes*. *Some of us stayed at home and some would come and work*. *So we used to work in turns (meaning shift work)*. *I work for a week then the other week*, *another person comes in to work” (LF*, HF05).

*“We had to bear with it (Meaning workload) only that sometimes we would shift them such that those working from home would at times come to work from the facility because we were still facing the work of the backlog*. *So they would work in shifts*. *In a week they would work three days*, *instead of five days*. *Then they would work from home for the two days” (LF*, HF02).

## Discussion

This study shows that several measures were employed to deliver HIV services during the COVID-19 pandemic restrictions in Kampala, Uganda. The measures ranged from the use of mobile phone technology to the adoption of novel client-centered ART delivery models, stepping up the use of existing community ART delivery models to the reorientation of health facility functioning to the COVID-19 pandemic restrictions, Although the context dictated the type and number of measures used to deliver HIV services amidst the COVID-19 pandemic restrictions, the measures described were mentioned by the participants at all the health facilities almost in equal occurrence. However, the most predominant measures included the use of mobile phone technology, use of motorcycle taxis, multi-month dispensing of ARVs, and ART delivery approaches namely, fast-track, home, peer, community pharmacy, and community client-led models. These should be considered in the interpretation of the results.

In Uganda, at least seven in 10 households have a mobile phone [19]. Mobile phone technology is one of the components of the package of care for PLHIV per the Uganda Ministry of Health recommendations. It is used for ART adherence counseling and support, patient tracking, improved linkage, and sending reminders concerning follow-up of appointments. Mobile phone technology is also recommended for text messaging to support ART adherence [16]. Our finding regarding the use of mobile phone technology to support ART adherence counseling, psychosocial support, and patient reminders regarding clinic appointments and referrals is therefore consistent with the Uganda Ministry of Health recommendations.

We found novel differentiated service delivery models, namely motorcycle taxis, and individualized ART delivery models were used to deliver ARVs. When the COVID-19 pandemic restrictions were imposed, some service providers entered into a strategic partnership with the Ugandan Ministry of Health (MOH) and the Infectious Diseases Institute (IDI) to deliver ARVs and condoms directly to the patient’s homes [20]. Our finding is consistent with this partnership. We found some patients desired to use private motorcycle taxis due to concerns around HIV status disclosure. No previous study has documented this evidence but the use of motorcycle taxis for ART delivery has been reported among patients at the IDI HIV clinic in Kampala, Uganda [21]. There is a need to sustain these measures developed by the IDI beyond the pandemic period in the delivery of HIV care although how and when this can be done, and whether it is possible remains questionable.

We found differentiated service delivery models for ART refills such as home-based refills, peer refills, fast-track refills, community pharmacy, community client-led ART delivery, and multi-month dispensing were stepped up during the restrictions. These offered patients different ways of continuing with HIV care and alignment with their needs and preferences [16]. Previous studies have reported that differentiated service delivery models ensure near-perfect ART adherence [22] including viral load suppression and retention [23]. Elsewhere [24], differentiated service delivery models have been reported as appropriate options for HIV treatment in sub-Saharan Africa during the COVID-19 lockdown. Fast-track ARV refills enabled the pick-up of medications from dispensing points and pharmacies [16]. Multi-month dispensing of ARVs ensured sufficient doses of ARVs during the restrictions and is consistent with the recommendations of the Uganda HIV treatment guideline [16]. This approach reduced frequent clinic visits which were either impossible or difficult during the restrictions and is consistent with a need to accelerate differentiated service delivery models for HIV care in sub-Saharan Africa during the lockdown [24] as well as the findings of a previous study [25].

Our findings regarding the reorientation of health facility functioning to the COVID-19 pandemic restrictions by encouraging and linking patients to access HIV services at the nearest health facility are among the novel measures which ensured sustained access to ARV refills, viral load monitoring, and psychosocial support. This measure has so far been reported by one study conducted among people with HIV and hypertension co-morbidity in Kampala, Uganda [13]. The study reported that access to HIV medicines remained nearly universal during the restrictions, with 49–66% of the patients with missed clinic appointments seeking care at the nearest health facility. Another reorientation of health facility functioning to the COVID-19 pandemic restrictions was the creation of new workspaces at health facilities to prevent the transmission of COVID-19 between healthcare providers and patients and a revised workflow to reduce patient waiting time. In addition, the transportation of healthcare workers to health facilities alongside the introduction of flexible work schedules and shift work enabled healthcare workers to provide HIV care throughout the restrictions. In particular, the transportation of health workers is consistent with the findings of a previous study at the IDI HIV clinic in Kampala, Uganda [21].

### Implications of study findings and lessons learned

Our findings have ramifications for HIV clinical practice, policy, and research. We have reported several measures for continuity of HIV care, both new and scale-up of already known/existing measures. Some of the measures can be adopted for delivering routine HIV care through new policy guidelines. Another important consideration is that several of the measures could be combined to form a package of high-impact practices and then investigated for effectiveness through interventional studies. This would generate reliable evidence for wider adoption of the measures including the addition to the HIV treatment guideline. Lastly, the measures could be considered for the delivery of HIV care in similar resource-constrained settings in developing countries.

Our findings highlight four major lessons. First, we learned that there is no functional health system without the existence of a resilient health workforce. Amidst the restrictions, healthcare workers persisted to provide HIV care even when the epidemiology (transmission, prevention, and risk factors) of the pandemic was unknown, possibly due to the desire to serve humanity. Therefore, we learned that the efficient delivery of HIV care requires a strong health system with sufficient size (number) and equitable distribution of dedicated healthcare workers who have adequate training, skills, and competence among others consistent with the WHO recommendations for health systems strengthening [26]. Second, we have learned that digital health technologies are critical for a strong health system. It became apparent that the health system should embrace digital health technologies to ensure universal health coverage. The use of mobile phone technology dramatically changed the delivery of HIV care amidst the restrictions because it supported the continuity of counselling, ART adherence, reminders about clinic appointments, and patient tracking amongst others. Third, we learned that patients understand the healthcare delivery measures that work for themselves. Patients got involved in shaping the decisions concerning the options for delivery of HIV care, treatment, and support. Unlike healthcare professionals who often operated from the expert viewpoint, patients’ understand their challenges, concerns, and the context within which service delivery happens. The pandemic revealed to us that there is a need for greater involvement of patients in conceptualizing, developing, and designing HIV care delivery options. Lastly, we learned that the measures for HIV service delivery should be context-oriented. Most of the measures for service continuity followed the issuance of guidance from the WHO and the Uganda Ministry of Health mandating health facilities to adopt measures to deliver essential health services amidst disruptions. With health facilities opting for different measures relevant to their context, we learned that there is no one golden standard measure for the delivery of HIV care. Similar health problems in different contexts might require different strategies.

### Study strengths and limitations

Our study has several strengths. First, to the best of our knowledge, this is one of the few studies which examined measures for continuity of HIV services during the COVID-19 pandemic restrictions in Uganda. We collected rich data from knowledgeable key informants, namely ART focal persons and lay health workers (peer mothers and linkage facilitators) who were engaged in the provision of HIV care, treatment, support, and prevention services and who had a better understanding of the functioning of the health system during the COVID-19 pandemic restrictions. However, there are limitations to the study. Our study was conducted in a predominantly urban population so there is a possibility that different measures might have been used in rural settings. Another concern is the possibility of recall and reporting biases because the restrictions were enforced nearly two years ago. However, this limitation was minimized by probing questions and restricting the interviews to participants who had worked at the respective clinics during the COVID-19 pandemic restrictions. Our study did not have data regarding measures for HIV prevention and HIV testing and counseling services during the COVID-19 pandemic restrictions, possibly because these services were not provided at the time.

## Conclusion and recommendations

Our study found that the health system adopted new measures to deliver HIV care, treatment, and support services. There was also the scaled-up of existing ART delivery models to ensure continuity of HIV services during the COVID-19 pandemic restrictions in Kampala, Uganda. We recommend that future studies, whether interventional or implementation sciences research, should logically map the different strategies to the needs of PLHIV and then examine whether and how they change the experiences of PLHIV including the effect on outcomes of HIV care. Certainly, such studies should be conducted before the measures are scaled up in the post-COVID-19 era.

## Supporting information

S1 File Interview guide. (PDF)

S2 File Final codebook. (DOCX)
