# Supplementary material for: Adaptations to HIV services delivery amidst the COVID-19 pandemic restrictions in Kampala, Uganda: A qualitative study
Source: PLOS Glob Public Health. 2022 Aug 23;2(8):e0000908. doi: 10.1371/journal.pgph.0000908 (PMC10022311; doi:10.1371/journal.pgph.0000908)
Supplement: S1 File — (PDF) [file pgph.0000908.s001.pdf]

13.1.15.1. APPENDIX I: KII guide for HIV/ART Focal Person, Linkage Facilitators, and peer mothers

Introduction: Thank you for accepting to participate in this interview. The focus of this interview is to discuss measures that have been used to ensure continuity of HIV services during and after the COVID-19 pandemic restrictions. I will ask you to describe how HIV services have been re-oriented during and after the pandemic; how existing community and patient-centered HIV delivery models have been modified to ensure continuity; staff alignment or re-deployment to ensure service continuity; modifications in the organization and delivery of HIV supplies and commodities; and any other HIV specific changes within the health facility.

Topic I: HIV services orientation

The COVID-19 pandemic restrictions have led to disruption in HIV services delivery at most health facilities. Tell me what measures were taken by the health facility at that time and now to ensure people living with HIV continue to access services.

- *Probe on measures to ensure HIV testing is done; measures to ensure people newly diagnosed with HIV are linked to care and started on treatment; measures to ensure people on HIV treatment remain in care; measures to ensure access to HIV viral load testing; specific measures for children, adolescents, and adults.*

Topic 2: Community and patient-centered HIV delivery models

During the restrictions, the delivery of HIV services in the community was equally affected. Share with me some of the measures that the health facility implemented or is implementing to ensure that people living with HIV continue to access care.

- *Probe on new models for ART delivery; new approaches to ongoing psychosocial and ART adherence support; approaches for reaching specific individuals in need of immediate help.*

Topic 3: Staff alignment or re-deployment to ensure service continuity

I would like to ask you about specific changes related to staffing. Please tell me of any changes that have been made in the organization of staffing at the health facility and community levels to ensure continuity of HIV care

- *Probe on changes in staff deployment and effect of this on HIV services delivery; changes in staff rooster/schedules and how it affected HIV service delivery.*

INFECTIOUS DISEASES INSTITUTE
RESEARCH ETHICS COMMITTEE (IDI-REC)
APPROVED
VALID UNTIL
30 JUL 2022

Topic 4: Modifications in the organization and delivery of HIV supplies and commodities

Let's talk about supplies and commodities for HIV care. Please tell me of any changes that were implemented or are being implemented to ensure a consistent supply of ARVs and HIV test kits.

- *Probe on changes in the monitoring of stock levels; changes in the ordering and distribution/re-distribution of HIV commodities or supplies; new approaches to dispensing such as multi-month dispensing, peer-led, etc.*

INFECTIOUS DISEASES INSTITUTE
RESEARCH ETHICS COMMITTEE (IDI-REC)
APPROVED
VALID UNTIL
30 JUL 2022
