# Supplementary material for: Adaptations to HIV services delivery amidst the COVID-19 pandemic restrictions in Kampala, Uganda: A qualitative study
Source: PLOS Glob Public Health. 2022 Aug 23;2(8):e0000908. doi: 10.1371/journal.pgph.0000908 (PMC10022311; doi:10.1371/journal.pgph.0000908)
Supplement: S2 File — (DOCX) [file pgph.0000908.s002.docx]

**S2 File: Final codebook**

| **Themes** | **Sub-themes** | **Description** | **Example** |
| --- | --- | --- | --- |
| Leveraging the use of mobile phone technology for HIV care | Mobile-phone-based ART adherence support. | Describes the use of mobile phones to support ART adherence. | *“We used to counsel them. It never stopped. The ones with high viral load we were counselling them on phone but for new patients who just tested, it was face to face counselling. We would counsel them immediately then we would start them on ART” (LF, HF06).* |
|  | Mobile-phone-based psychosocial counseling. | Describes the use of mobile phones for psychosocial counseling. | *“We would make phone calls and we conduct an online counselling session. So we would get on phone and tell them the importance of adhering well. And the proper way of taking the medication through phone. We had them counselled through phone” (LF, HF04).* |
|  | Mobile-phone-based reminders about clinic appointments and referrals. | Describes the use of a mobile phone for reminding PLHIV to turn up for clinic appointments. | *“Another thing we used to do was to pre-call and remind them (PLHIV) about their appointments. So those who could afford used to come to the facility” (LF, HF06).* |
| Adoption of novel differentiated service delivery models for ART delivery | Use of motorcyclists to deliver ARVs. | Describes access to ARVs using motorcycle taxis. | *“During COVID time (meaning the COVID-19 pandemic restrictions), we had the boda boda delivery model, which was not there before. It was introduced during that period. Boda boda model worked in a way that when a patient's appointment date reached and they could not reach the facility because of the lockdown and the transport issues, IDI (Infectious Diseases Institute) came in with the model of boda boda. So we could just send the drugs to our patients to their homes. Because they were trying to limit the numbers so that we have few patients at a time” (LF, HF02).* |
|  | Individualized ART delivery for patients with non-disclosed HIV status. | Describes approaches to delivering ARVs according to individual needs. | *“Some patients, you just have to understand them. They have too much stigma. They say, "You don't send us your boda boda (meaning motorcyclists to deliver ARVs). I will get another boda boda and pay for it myself", because he will not know what he is carrying” (LF, HF05).* |
| Scale-up of existing differentiated service delivery models for ART delivery | Multi-month dispensing of ARVs. | Describes an ART delivery approach where PLHIV received ARVs for a longer period compared to the standard of care. | *“During the lockdown, the months for refiling ARVs [meaning anti-retroviral drugs) had to increase at least from 2-3 months or even more. Those not due for viral load testing were getting more than four months refill” [LF, HFO2].* |
|  | Fast-track ARV refills. | Describes an ART delivery approach where PLHIV were refilled ARVs without delay provided they did not need to see a clinician (nurse, medical doctor, or any other clinician). | *“So all we were doing was refill and go, refill and go, refill and go. So at that time we were not differentiating care. So once you come in, we give you three months, you walk away, come in, three months, and walk away ( FP, HF04).* |
|  | Home-based ARV refill. | Describes an approach to ART delivery where ARVs were delivered through home visits. | *However, there are those ones that were like for me, I stay nearby but I cannot come to the health facility. So, what we (HIV Care Team) used to do was to get a locator form from the file and then try our level best to pick the drugs (meaning ARVs), inform the clinician, then sign in the client's file and then carry them (meaning the ARVs) to the client's home. That’s how we used to do it.” (PM, HF04).* |
|  | ART delivery approach. | Description of ART delivery approach where peers, mostly PLHIV who knew each other, delivered ARVs to fellow peers. | *“The expert clients would call the patients and if they were unable to come for the drugs, they would reach there and take for them the drugs” (FP, HF04).* |
|  | Use of community pharmacy | Describes the approach to ART delivery where ARVs were taken to the community where PLHIV lived and worked. | *“We have clients here who get drugs from the community pharmacy. So those ones used to get drugs from the community pharmacy. Apart from the community pharmacy and boda boda, there was nothing else” (LF, HF02).* |
|  | Use of Community Client-led ART delivery model. | Describes an approach to ART delivery where people living with HIV formed a group of 10 people, with one of the group members collecting ARVs for all the members. The responsibility to collect ARVs from the health facility is rotational among the members. | *“So they make a group of six people who reside from the same community. But before they start their group, they first come here then you orient them in doing their work. Then in that group, we decide who will come first. Now you give them appointments every two months. You give them appointment every two months then they alternate, today it is patient A after two months it is patient B after two months it is patient C like that. So when they pick the drugs, they go back to the community and give to the other group members. But when patient A comes you retrieve files for all the group members.” (HF01, LF).* |
| Reorientation of health facility functioning to the COVID-19 pandemic restrictions. | Use of nearby health facilities for ARV refill and viral load monitoring. | An approach to continuity of care where PLHIV collected ARVs from any nearby health facility. | *“Those ones who were not within our proximity, we would tell them to go to health facilities near them and we would call the other facility to ensure their retention” (LF, HF04).* |
|  | Transportation of health workers to health facilities and flexible work schedules. | An approach to HIV care where healthcare workers were provided with a private transportation system to and fro their home to the health facility, including working at the nearby health facility. | *“We were coming everyday although the staff was reduced. Those ones coming from far could not come because of transport. The few who were supposed to come, there were those who would be picked and dropped here by car and then there were those who could not.” (PM, HF02).* |
|  | Introduction of shift work. | An approach to ensure continuity of HIV care where healthcare providers worked on alternate days and/or hours. | *“Yes, there were changes. Some of us stayed at home and some would come and work. So we used to work in turns (meaning shift work). I work for a week then the other week, another person comes into work” (LF, HF05).* |
